# Enhanced Breakdown Voltage in GaN HEMTs Using Step-Graded AlGaN Buffer Layers

**DOI:** 10.3390/mi17101141

**Published:** 2026-09-30

**Authors:** Junchun Bai, Xu Liu, Hongchang Tao, Huake Su, Jincheng Zhang, Yue Hao

**Affiliations:** State Key Laboratory of Wide-Bandgap Semiconductor Devices and Integrated Technology, Xidian University, Xi’an 710071, China; xdbaijunchun@163.com (J.B.); xliu23@stu.xidian.edu.cn (X.L.); taohongchang@xidian.edu.cn (H.T.); huakexidian@126.com (H.S.); yhao@xidian.edu.cn (Y.H.)

**Keywords:** GaN HEMT, step-graded AlGaN buffer, breakdown voltage

## Abstract

In this work, step-graded AlGaN buffer layers were introduced into GaN high-electron-mobility transistors to enhance their off-state voltage-blocking capability without intentional deep-level buffer doping. Hall measurements showed increased two-dimensional electron gas sheet densities, while the electron mobilities remained above 1550 cm^2^/V·s. The fabricated devices exhibited similar threshold voltages near −18 V and comparable pulsed output current densities. At approximately 1000 V, sample B showed an average drain leakage-current reduction of approximately 27.5% relative to sample A. The average breakdown voltage increased from 1540 V for the conventional GaN-buffer device to 1814 V for the optimized step-graded AlGaN-buffer device, corresponding to an enhancement of approximately 17.8%. TCAD simulations show that the step-graded buffer introduces a multi-step electron-blocking barrier and enhances vertical carrier confinement. Under high-voltage off-state conditions, it also redistributes the drain-side electrostatic potential and suppresses electron penetration into the buffer. In addition, dislocation-related trapping may also contribute to the improved off-state blocking performance. These results demonstrate that step-graded AlGaN buffers offer an effective approach for improving the breakdown performance of GaN HEMTs while largely preserving their conduction capability.

## 1. Introduction

Gallium nitride (GaN) high-electron-mobility transistors (HEMTs) have emerged as leading candidates for next-generation power electronics, owing to their wide bandgap, high critical electric field, and superior two-dimensional electron gas (2DEG) transport properties [1,2]. For high-voltage power switching applications, breakdown voltage (*V*_BR_) is a critical figure of merit that directly determines the maximum power handling capability and operating voltage range of the device [3]. However, the experimentally achieved *V*_BR_ in GaN HEMTs often falls substantially below the theoretical limit predicted by the GaN material system [4]. This performance gap is largely associated with premature breakdown induced by multiple leakage paths, among which buffer leakage is an important limiting factor [5,6]. Buffer leakage arises from electron injection from the channel into the underlying buffer layer under high drain bias, followed by transport through the buffer to the substrate or directly to the drain electrode. These injected electrons can also be captured by deep-level traps, leading to dynamic on-resistance degradation and current collapse that compromise device reliability [7]. Substantial efforts have been devoted to suppressing buffer leakage and enhancing breakdown voltage. Carbon and iron doping are widely employed to introduce deep-level acceptor-like traps that compensate residual background carriers and increase buffer resistivity [8,9,10,11]. Back-barrier structures utilizing AlGaN or InGaN layers have also been demonstrated to improve electron confinement by increasing the conduction band offset beneath the channel [12,13,14]. However, carbon-related deep levels may induce charge trapping, current collapse, and dynamic on-resistance degradation [15]. Iron doping may also introduce a pronounced memory effect during epitaxial growth, increasing the risk of residual Fe incorporation and cross-contamination in subsequent layers [16]. Such trade-offs limit the effectiveness of these approaches in achieving high breakdown voltage without sacrificing dynamic performance or process stability. Buffer-layer engineering provides an alternative approach to tailoring both the structural and electrical properties of GaN heterostructures. Previous studies have shown that epitaxial buffer design and growth conditions can significantly influence strain relaxation, crystalline quality, and dislocation behavior in III-nitride heterostructures [17,18]. In particular, step-graded AlGaN buffers have been employed in GaN-on-Si heteroepitaxy to regulate strain and improve structural quality [17]. Experimental studies have shown that a three-step AlGaN buffer can reduce leakage current compared with a two-step structure in AlGaN/GaN HEMTs on Si [19]. However, experimental studies on thin step-graded AlGaN buffers on sapphire for kilovolt-class GaN HEMTs are still limited.

In this work, we demonstrate GaN HEMTs incorporating step-graded AlGaN buffer layers for enhanced off-state breakdown voltage. Compared with the conventional GaN-buffer structure, the step-graded AlGaN buffer introduces a multi-step conduction-band profile beneath the channel, which reduces electron injection into the buffer under high drain bias. The broader HRXRD (102) rocking curves of the step-graded samples indicate increased edge-dislocation-related structural disorder, which may contribute to electron trapping under high-field conditions. The average breakdown voltage increased from 1540 V for the conventional GaN-buffer HEMT to 1814 V for the optimized step-graded AlGaN-buffer HEMT, corresponding to an enhancement of approximately 17.8%. This work suggests that step-graded AlGaN buffers provide an effective route to improving the voltage blocking capability of GaN HEMTs without relying on intentional deep-level buffer doping.

## 2. Materials and Methods

**Figure 1:**
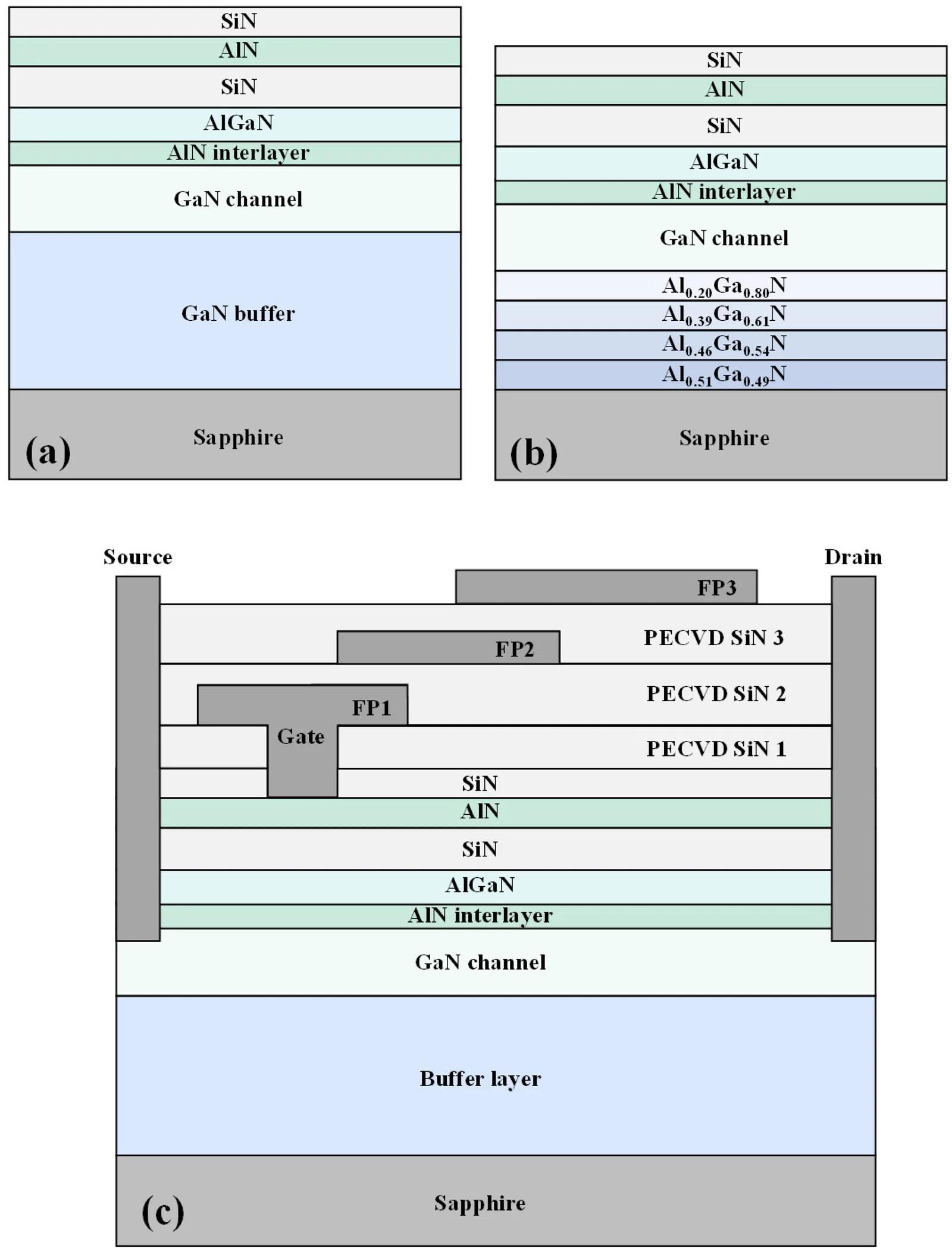
Schematic illustrations of (**a**) the conventional GaN-buffer epitaxial structure, (**b**) the step-graded AlGaN-buffer epitaxial structure, and (**c**) the fabricated HEMT device structure.

Figure 1a,b show the schematic epitaxial structures grown on sapphire substrates by metal–organic chemical vapor deposition. Four samples with different buffer designs were prepared. Except for the buffer layer, all samples have the same epitaxial structure, consisting of a 30-nm AlN nucleation layer, a 300-nm high-temperature GaN channel layer, a 1-nm AlN insertion layer, a 22-nm Al_0.23_Ga_0.77_N barrier layer, and a 45-nm SiN/7-nm AlN/7-nm SiN in-situ passivation stack. The 45-nm SiN layer serves as the main passivation and gate dielectric layer, the 7-nm AlN layer acts as an etch-stop layer for gate-recess control, and the upper 7-nm SiN layer protects the AlN layer during subsequent processing. Additional SiN was later deposited by Plasma-Enhanced Chemical Vapor Deposition (PECVD) for further device passivation and dielectric isolation. Sample A employs a 600-nm GaN buffer layer as the reference structure. Although thicker high-resistivity GaN buffers are generally advantageous for high-voltage blocking, the 600-nm GaN buffer provides a process-matched reference for evaluating the step-graded AlGaN buffer without introducing a large difference in total buffer thickness. It is a mature and stable conventional structure in our growth and device platform. Samples B, C, and D were designed with step-graded AlGaN buffer layers. The step-graded AlGaN buffer contains four AlGaN layers with Al compositions of 0.51, 0.46, 0.39, and 0.20. For sample B, the thicknesses of the Al_0.51_Ga_0.49_N, Al_0.46_Ga_0.54_N, Al_0.39_Ga_0.61_N, and Al_0.20_Ga_0.80_N layers are 25 nm, 35 nm, 50 nm, and 70 nm, respectively. The corresponding thicknesses are 25 nm, 35 nm, 75 nm, and 105 nm for sample C, and 25 nm, 35 nm, 100 nm, and 140 nm for sample D.

All samples were fabricated using the same device process. An additional 140-nm-thick PECVD SiN dielectric layer was first deposited on the in-situ SiN/AlN/SiN passivation stack. The dielectric in the isolation regions was then etched to a depth of approximately 147 nm, followed by ion implantation to define and electrically isolate the active regions. For the source and drain contacts, ohmic contact windows were etched to a depth of approximately 226 nm. A Ti/Al/Ti metal stack of 40/200/30 nm was subsequently deposited and annealed at 575 °C for 120 s to form ohmic contacts. The dielectric in the gate region was then etched to a nominal depth of approximately 147 nm, removing the 140-nm PECVD SiN and the upper 7-nm in-situ SiN protective layer while leaving the 45-nm SiN/7-nm AlN stack beneath the gate. The gate electrode and first gate-connected field plate (FP) were then formed using Ti/Al/Ti metallization of 45/340/30 nm. After deposition of a 250-nm-thick SiN dielectric layer, contact vias were opened and the second gate-connected field plate was formed using Ti/Al/Ti (45/340/30 nm). A further 350-nm-thick SiN dielectric layer was then deposited, followed by via opening and the formation of the third gate-connected field plate using Ti/Al/Ti (100/1500/30 nm). Finally, a 1-μm-thick SiN passivation layer was deposited, and the contact-pad openings were formed. Figure 1c schematically illustrates the common HEMT device structure used for all four samples. The fabricated HEMTs have a gate length of 2 μm, a gate–source spacing of 4 μm, a gate–drain spacing of 18.5 μm, and a source–drain spacing of 24.5 μm. The devices consist of 44 gate fingers with a width of 300 μm per finger, corresponding to a total gate width of 13.2 mm.

High-resolution X-ray diffraction measurements were performed using a Bruker D8 Discover diffractometer to evaluate the crystal quality of the samples. Raman spectra were recorded using a Jobin Yvon LavRAM HR800 spectrometer (HORIBA Jobin Yvon S.A.S., Villeneuve d’Ascq, France) with a 633 nm excitation laser to evaluate the residual stress state. Hall-effect measurements were performed using an HL9900 Hall measurement system (Toho Technology Inc., Chicago, IL, USA) to determine the sheet carrier density and electron mobility. The I-V characteristics of the devices were investigated using a Keysight B1500A semiconductor parameter analyzer and B1505A power device analyzer (Keysight Technologies, Inc., Santa Rosa, CA, USA).

## 3. Results and Discussion

**Figure 2:**
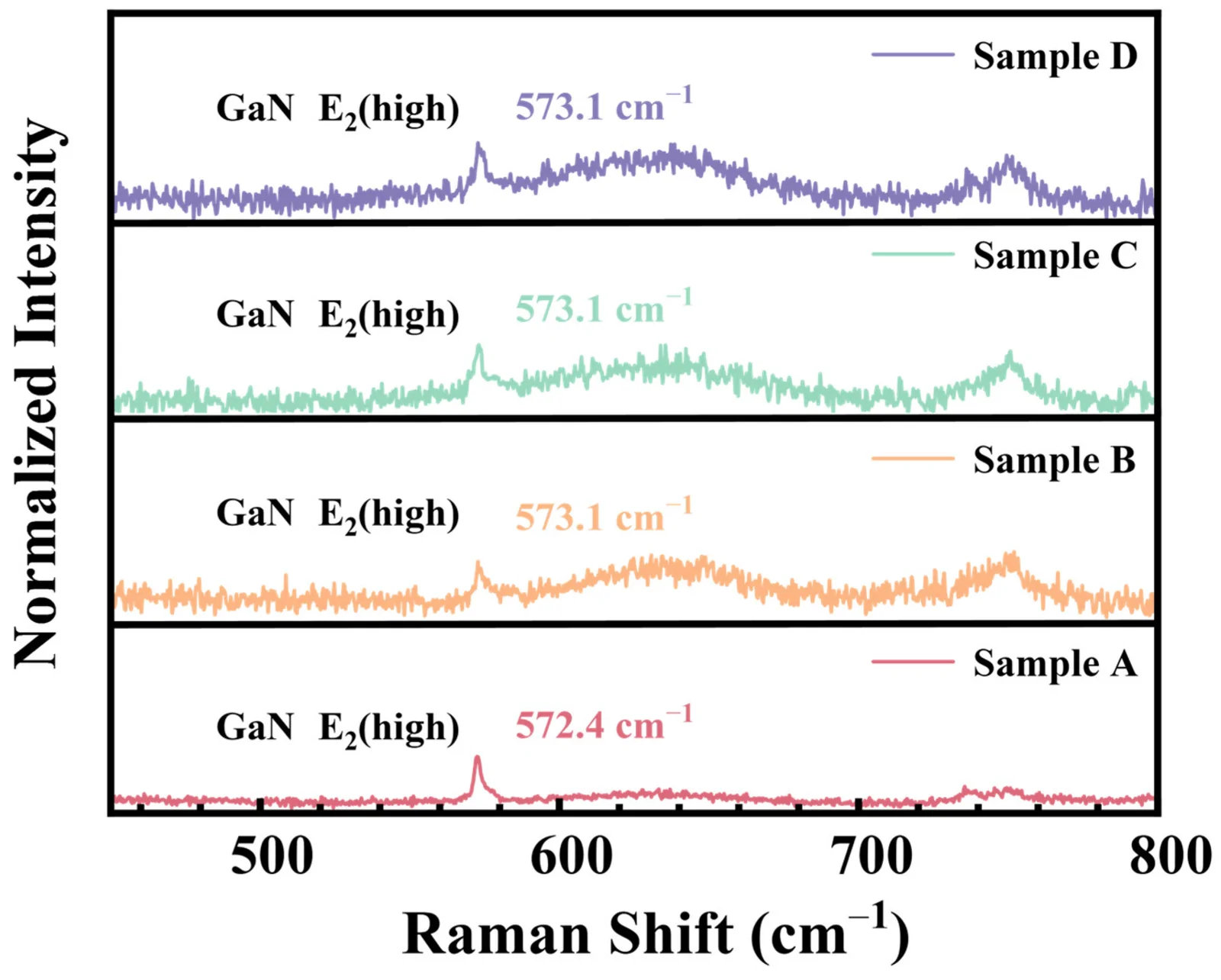
Raman spectra of samples A, B, C, and D.

Raman spectroscopy was employed to evaluate the residual stress state of the samples, as shown in Figure 2. The GaN E_2_(high) phonon peak of sample A is located at 572.4 cm^−1^, while those of samples B, C, and D shift to 573.1 cm^−1^. The slight blue shift of the GaN E_2_(high) phonon peak suggests that the step-graded AlGaN buffer introduces a slightly stronger compressive stress compared with the conventional sample. This result is attributed to the lattice mismatch between the step-graded AlGaN buffer and the subsequently grown GaN layer. The smaller in-plane lattice constant of AlGaN results in a stronger compressive strain in the GaN layer.

**Figure 3:**
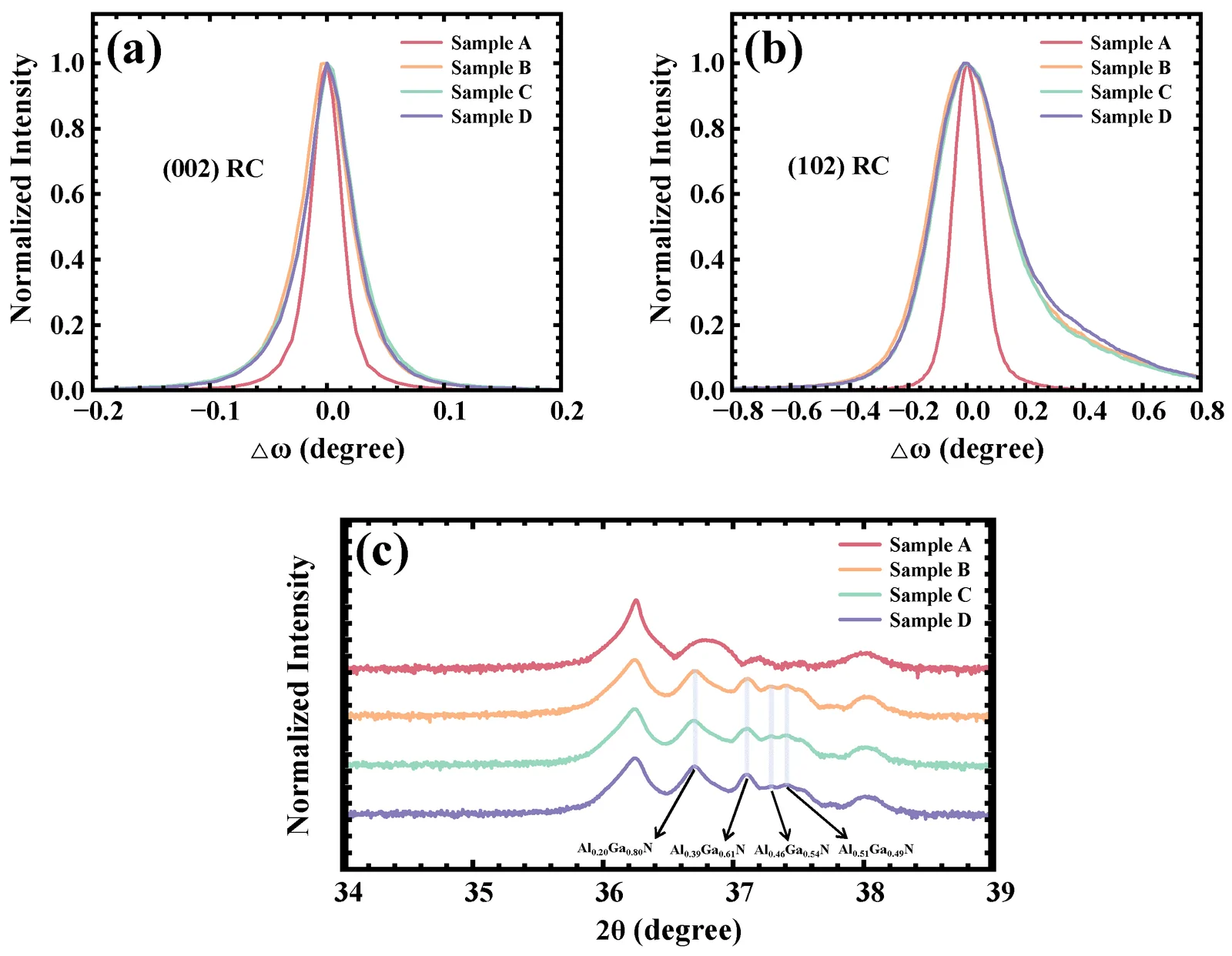
X-ray diffraction characterization of samples A, B, C, and D: GaN (**a**) (002) and (**b**) (102) rocking curves, and (**c**) symmetric (004) ω–2θ scans.

HRXRD was further employed to evaluate the crystal quality of the samples. The rocking curves of the (002) and (102) planes for all samples are shown in Figure 3a,b. Sample A exhibits a full-width at half-maximum (FWHM) of 106.7 arcsec for the (002) plane and 416.8 arcsec for the (102) plane. For samples B, C, and D, the (002) FWHM values are 170.9 arcsec, 171.1 arcsec, and 168.9 arcsec, respectively, while the corresponding (102) FWHM values are 1090.4 arcsec, 1045.0 arcsec, and 1089.1 arcsec. In the GaN material, the FWHM of the (002) rocking curve mainly reflects the screw dislocation density, while the (102) rocking curve is more sensitive to edge dislocations [20,21]. The much broader (102) rocking curves of samples with step-graded AlGaN buffers indicate a higher edge dislocation density. This result originates from the lattice mismatch between the AlGaN buffer and the GaN channel layer. The lattice mismatch induces strain relaxation during epitaxy, which is partially accommodated by the formation of edge dislocations. Figure 3c shows the symmetric (004) ω–2θ XRD scans of samples A–D. Four distinct AlGaN diffraction peaks are observed in samples B-D, corresponding to the four step-graded AlGaN buffer layers. After considering the strain effect, the estimated Al compositions are approximately 0.20, 0.39, 0.46, and 0.51, in good agreement with the designed values.

Hall-effect measurements were performed to further evaluate the 2DEG transport properties. Sample A with the conventional GaN buffer shows a sheet carrier density of 1.09 × 10^13^ cm^−2^ and an electron mobility of 1817.3 cm^2^/V·s. For the step-graded AlGaN-buffer samples, sample B shows a sheet carrier density of 1.18 × 10^13^ cm^−2^ and a mobility of 1578.77 cm^2^/V·s, sample C shows 1.19 × 10^13^ cm^−2^ and 1555.17 cm^2^/V·s, and sample D shows 1.20 × 10^13^ cm^−2^ and 1558.32 cm^2^/V·s. The step-graded samples therefore exhibit a modest increase in the Hall-extracted sheet density together with a decrease in electron mobility. The change in the extracted sheet density may be influenced by both the modified polarization environment and changes in the Hall factor associated with carrier scattering. Previous studies on AlGaN/GaN heterostructures have shown that variations in scattering mechanisms can modify the Hall factor and consequently affect the carrier density extracted from Hall measurements [22]. The reduced Hall mobility is consistent with enhanced carrier scattering associated with the modified structural and interfacial environment, including increased edge-dislocation-related disorder and possible degradation of the interface quality [23,24]. The Raman, HRXRD, and Hall measurement results of samples A–D are summarized in Table 1.

**Figure 4:**
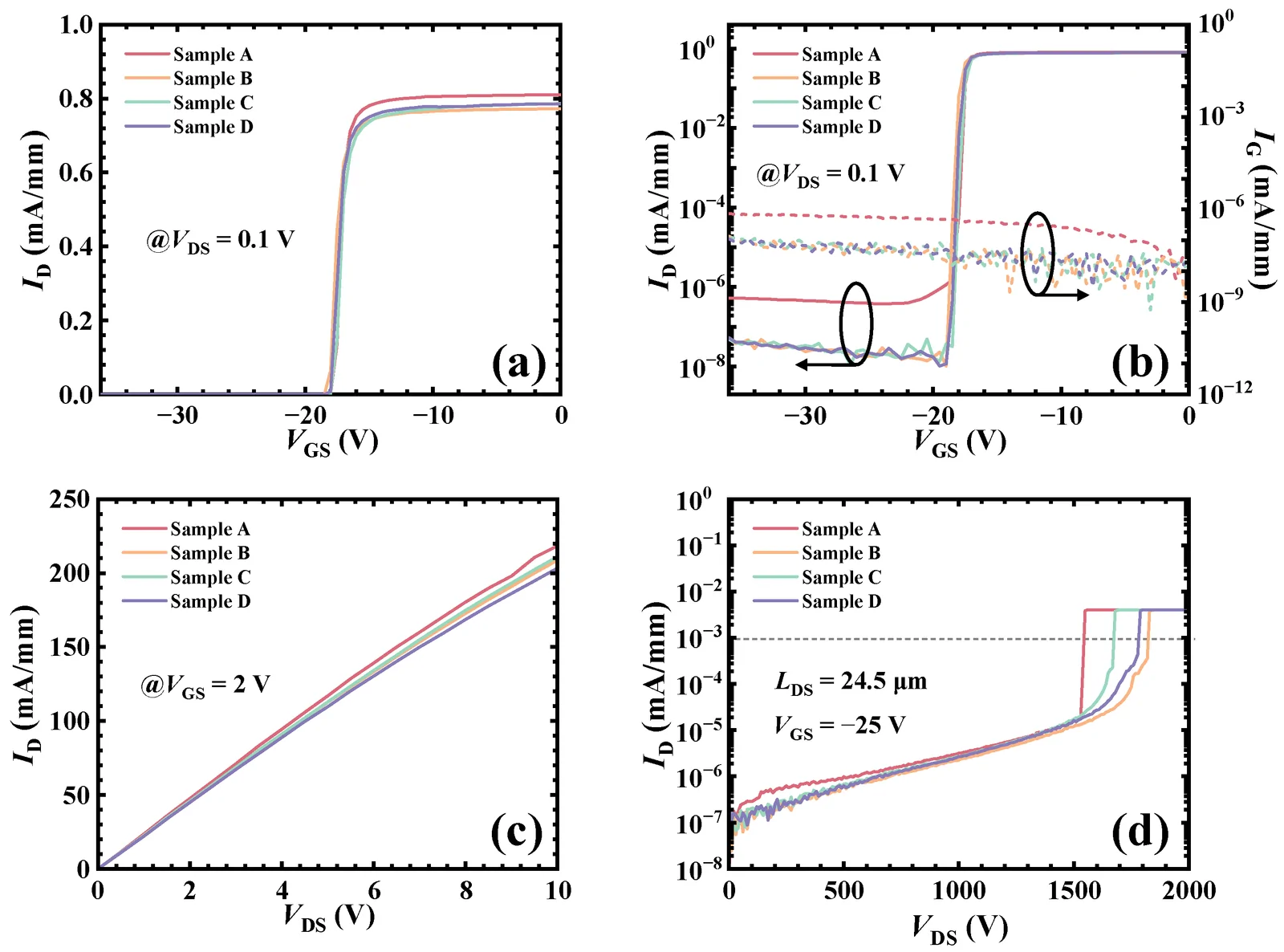
Transfer characteristics plotted in (**a**) linear and (**b**) logarithmic scales, and (**c**) pulsed output characteristics and (**d**) breakdown characteristics of samples A, B, C, and D. In (**b**), the solid and dashed lines represent the drain current and gate current, respectively.

**Figure 5:**
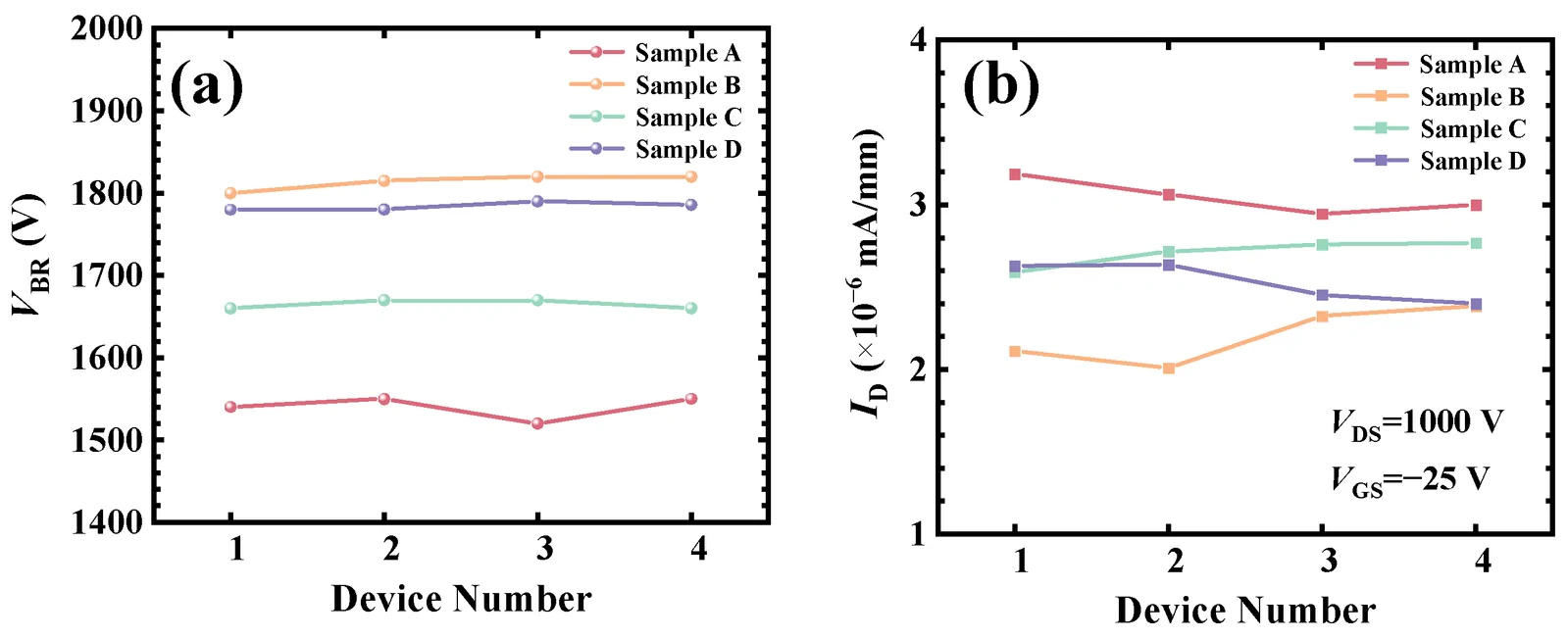
Device-to-device statistical distributions of (**a**) breakdown voltage and (**b**) drain leakage current at approximately 1000 V for samples A–D. Four devices were measured for each sample.

HEMT devices were fabricated on all samples, and their electrical characteristics are summarized in Figure 4. The transfer characteristics measured at *V*_DS_ = 0.1 V are plotted on linear and logarithmic scales in Figure 4a and Figure 4b, respectively. All devices exhibit depletion-mode operation and can be effectively pinched off under negative gate bias. The threshold voltages extracted by linear extrapolation are −17.9 V, −18.2 V, −17.9 V, and −18.0 V for samples A, B, C, and D, respectively. The relatively large equivalent dielectric thickness reduces the gate capacitance and requires a larger negative gate bias to deplete the 2DEG, which contributes to the threshold voltages near −18 V. All samples show comparable drain currents at *V*_GS_ = 0 V. The corresponding on/off current ratios are 1.56 × 10^6^, 1.57 × 10^7^, 1.64 × 10^7^, and 1.48 × 10^7^ for samples A, B, C, and D, respectively. Compared with sample A, the samples with the step-graded AlGaN buffer exhibit an approximately one-order-of-magnitude reduction in the off-state leakage current while maintaining comparable drain currents. The pulsed output characteristics are shown in Figure 4c. The measurements were performed with *V*_DS_ swept from 0 V to 10 V. A pulse width of 100 μs and a pulse period of 10 ms were used, corresponding to a duty cycle of 1%. At *V*_GS_ = +2 V and *V*_DS_ = 10 V, sample A shows a drain current of 218.4 mA/mm, while samples B, C, and D exhibit 208.7 mA/mm, 211.1 mA/mm, and 203.4 mA/mm, respectively. The step-graded AlGaN-buffer devices maintain pulsed output current densities above 200 mA/mm, indicating that their conduction capability is not severely degraded. The off-state breakdown characteristics are shown in Figure 4d. During the measurement, the source electrode was grounded, the gate voltage was fixed at *V*_GS_ = −25 V, and the drain voltage was swept from 0 to 2000 V with a step of 10 V. *V*_BR_ is defined as the maximum sustained drain voltage before abrupt breakdown or current-compliance termination during the high-voltage sweep. For the representative devices, samples A, B, C, and D achieve breakdown voltages of 1540 V, 1820 V, 1670 V, and 1780 V, respectively. The maximum enhancement reaches 18.2% for sample B. These results demonstrate that the step-graded AlGaN buffer significantly improves the voltage-blocking capability of the HEMTs. One possible contribution to the improved breakdown performance is the presence of dislocation-related acceptor-like states in the step-graded samples. The broader HRXRD (102) rocking curves indicate increased edge-dislocation-related structural disorder, which may be associated with additional acceptor-like states [25]. Under high drain bias, these states may capture or compensate injected electrons in the buffer, thereby suppressing leakage transport and contributing to an increased breakdown voltage. To evaluate device-to-device variation, four devices were measured for each sample. The statistical distributions of breakdown voltage and drain leakage current at approximately 1000 V are shown in Figure 5. The average breakdown voltages of samples A, B, C, and D are 1540, 1814, 1665, and 1784 V, respectively. Compared with sample A, sample B exhibits an average breakdown-voltage enhancement of approximately 17.8%. The corresponding average drain leakage currents are 3.05 × 10^−6^, 2.21 × 10^−6^, 2.71 × 10^−6^, and 2.53 × 10^−6^ mA/mm, respectively. Sample B thus exhibits an average leakage-current reduction of approximately 27.5% relative to sample A. The breakdown voltage does not increase monotonically with the total thickness of the step-graded AlGaN buffer. Changing the graded-layer thickness affects both the vertical electrostatic distribution and the crystalline properties developed during epitaxial growth. Sample C has a lower (102) FWHM than samples B and D, indicating less edge-dislocation-related broadening. If associated acceptor-like states contribute to high-field electron trapping, this difference may imply weaker trapping or charge compensation in sample C and could partly contribute to its lower breakdown voltage. Thus, optimization requires a trade-off among electrostatics, buffer thickness, and material properties rather than simply increasing buffer thickness.

**Figure 6:**
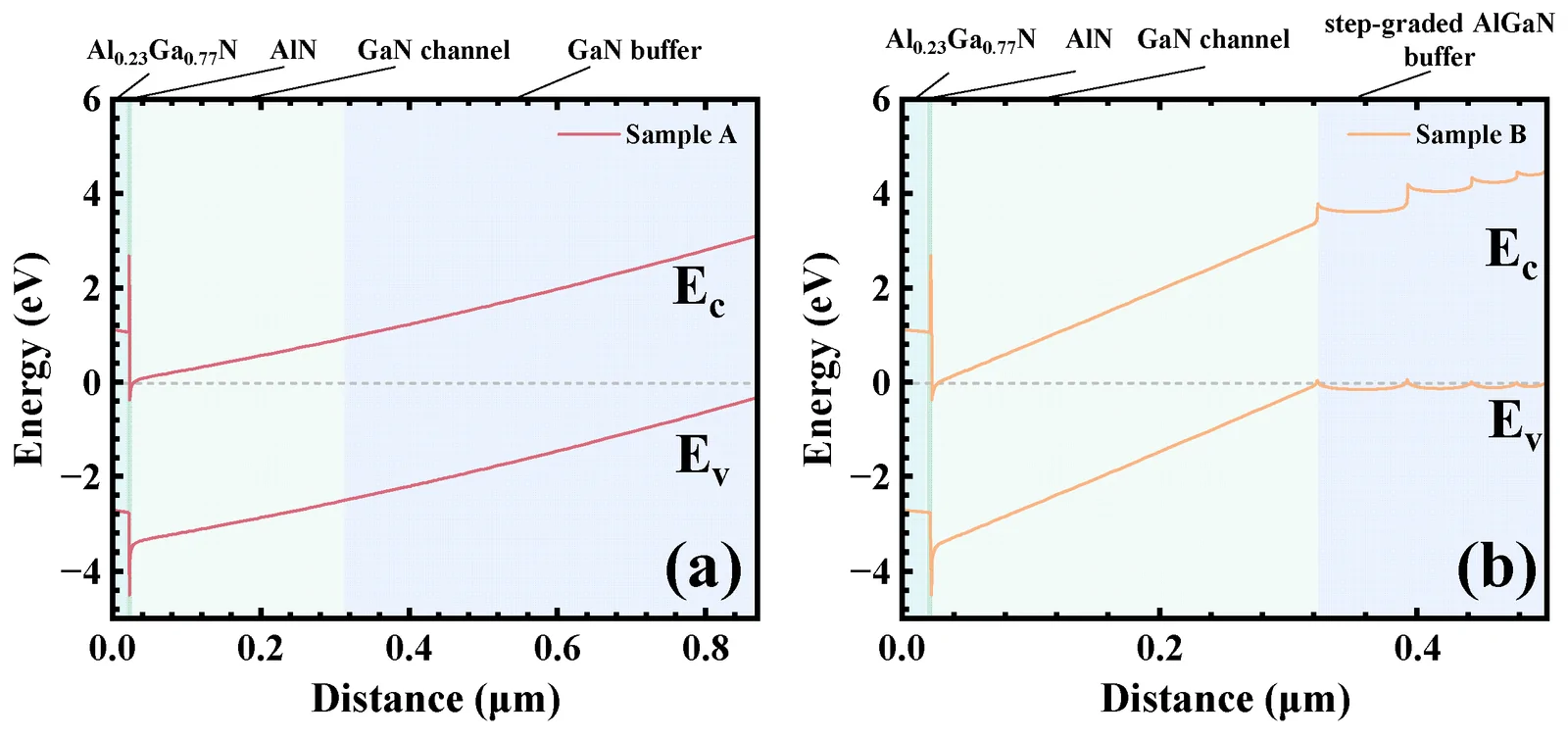
Simulated vertical energy-band profiles of (**a**) the conventional GaN-buffer structure and (**b**) the step-graded AlGaN-buffer structure. The gray dashed line represents the Fermi level.

**Figure 7:**
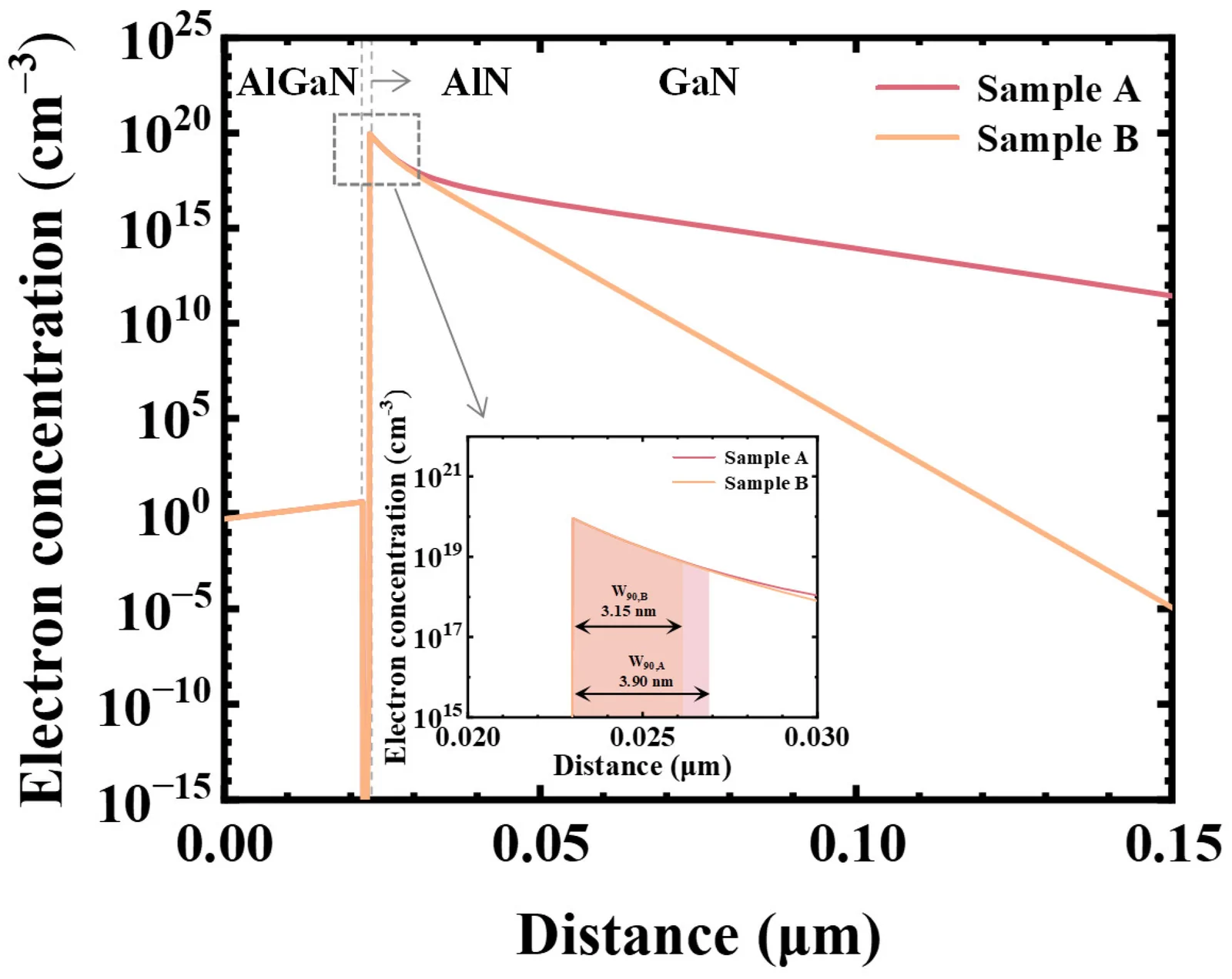
Simulated electron distributions near the AlN/GaN interface for samples A and B under thermal equilibrium. The inset shows the 90% electron-distribution widths, with *W*_90_ = 3.90 nm for sample A and *W*_90_ = 3.15 nm for sample B.

To investigate the mechanism underlying the enhanced breakdown voltage, TCAD simulations were performed for the conventional GaN-buffer and step-graded AlGaN-buffer structures. The simulations employed a drift–diffusion transport model with Fermi–Dirac statistics, Shockley–Read–Hall recombination, and field-dependent mobility. Strain-dependent spontaneous and piezoelectric polarization was included, and self-consistent Schrödinger–Poisson calculations were used to evaluate the equilibrium 2DEG distribution. Standard material parameters for GaN and AlGaN were adopted, with the AlGaN properties determined according to the corresponding Al compositions. Figure 6 shows the energy-band profiles under thermal equilibrium. Compared with the conventional GaN buffer, the step-graded AlGaN buffer forms a multi-step band profile beneath the channel. The different Al compositions of the AlGaN sublayers result in different bandgaps and electron affinities, producing band offsets at the heterointerfaces. In addition, the polarization difference between adjacent AlGaN layers introduces negative polarization charges at the interfaces, causing upward bending of both the conduction and valence bands. These band offsets and polarization-induced band bending form a step-like electron-blocking barrier in the buffer. The step-like barrier suppresses electron leakage from the channel toward the substrate. To further evaluate the influence of the step-graded AlGaN buffer on electron confinement, the electron distributions near the AlN/GaN interface were calculated, as shown in Figure 7. In both structures, the electrons are strongly localized near the AlN/GaN interface. However, compared with sample A, the electron distribution in sample B is more spatially confined, with the 90% electron-distribution width (*W*_90_) decreasing from 3.90 nm to 3.15 nm. Here, *W*_90_ is defined as the distance from the AlN/GaN interface containing 90% of the integrated electron density. These results indicate that the step-graded AlGaN buffer modifies the vertical electrostatic potential and strengthens the quantum confinement of electrons in the channel, thereby reducing their penetration toward the underlying buffer. The simulated 2DEG sheet densities are 1.17 × 10^13^ cm^−2^ and 1.13 × 10^13^ cm^−2^ for samples A and B, respectively, indicating comparable equilibrium carrier densities in the two structures. This result differs slightly from the Hall measurements, in which sample B exhibits a higher sheet density than sample A. Such a difference may partly arise from variations in the Hall factor caused by changes in the carrier-scattering environment. Previous studies on AlGaN/GaN heterostructures have shown that changes in scattering mechanisms can modify the Hall factor and consequently affect the Hall-extracted 2DEG density even when the actual carrier concentration remains nearly unchanged [22]. Therefore, the modest increase in the experimentally extracted Hall sheet density of sample B may not originate solely from an increase in the equilibrium 2DEG population. The GaN/Al_0.2_Ga_0.8_N interface beneath the 300-nm GaN channel introduces a conduction-band discontinuity and polarization-induced interface charge. This back-interface modifies the vertical electrostatic potential and contributes to electron confinement within the GaN channel. It may also reduce the penetration of electrons and drain-induced electrostatic potential into the underlying buffer, thereby suppressing buffer punch-through under high drain bias. For the present device geometry, conventional lateral short-channel effects are expected to be relatively weak because of the 2-μm gate length and 18.5-μm gate-to-drain spacing. Therefore, the influence of the bottom interface is mainly associated with vertical carrier confinement and buffer punch-through suppression.

**Figure 8:**
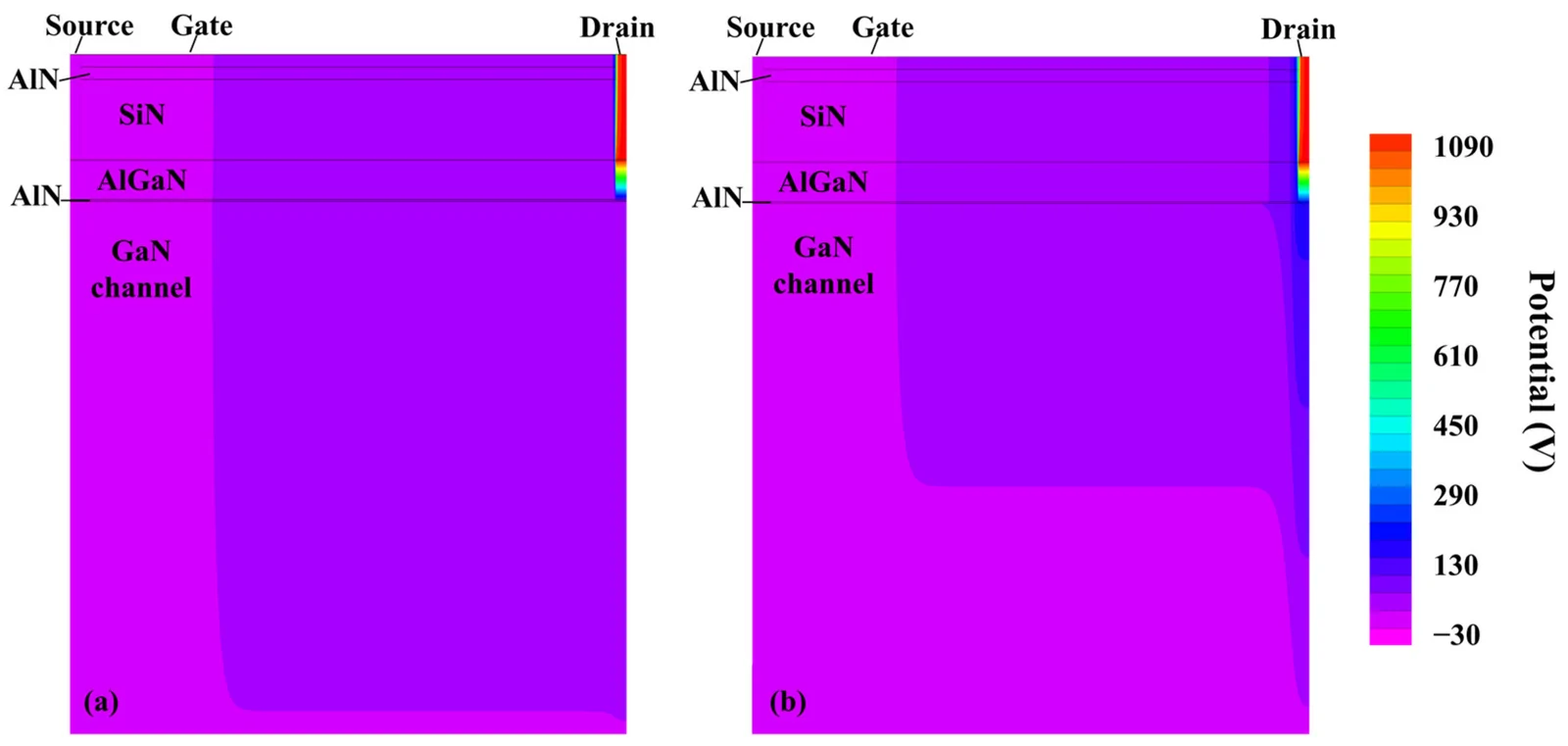
Simulated two-dimensional electrostatic-potential distributions of (**a**) sample A and (**b**) sample B at *V*_GS_ = −25 V and *V*_DS_ = 1100 V.

**Figure 9:**
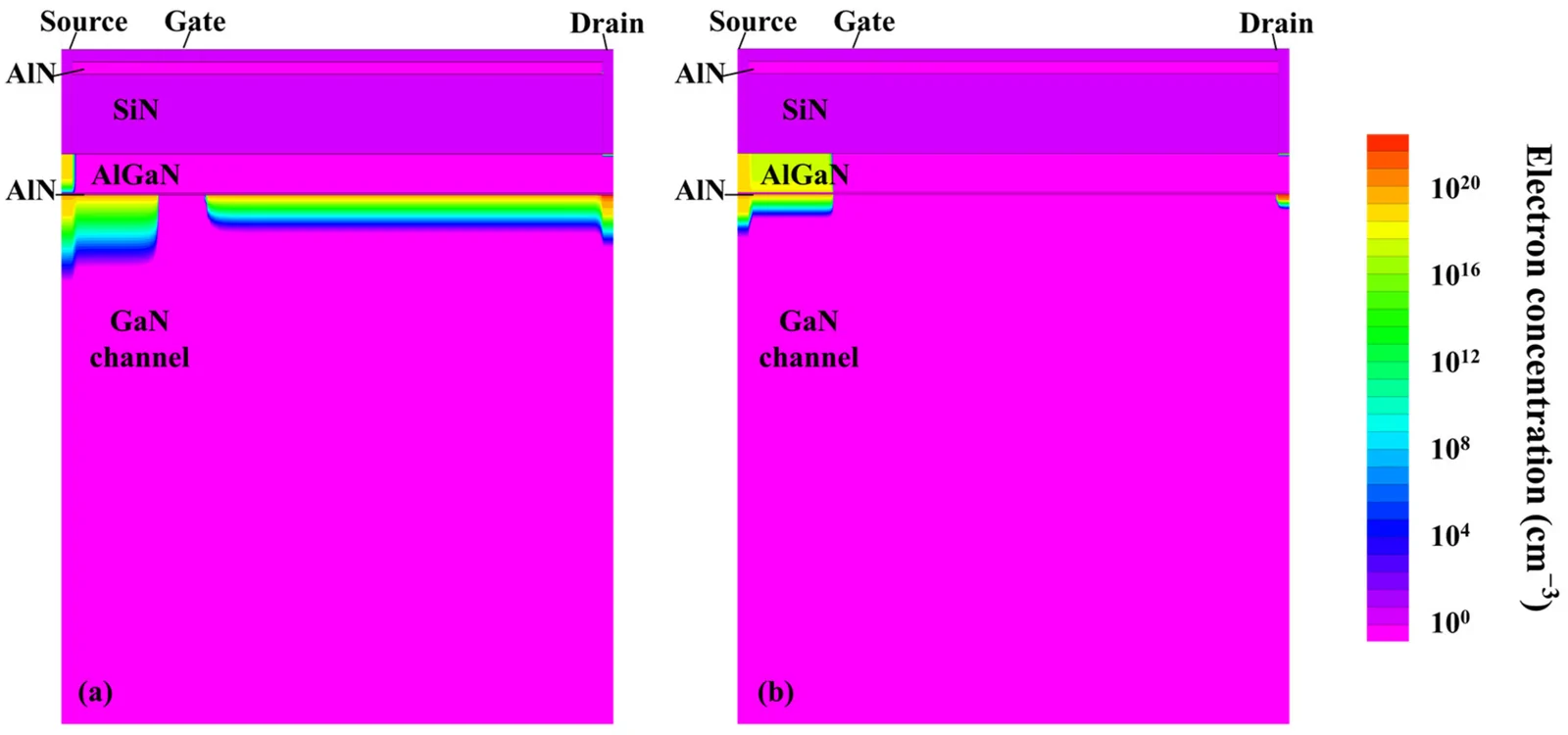
Simulated two-dimensional electron-concentration distributions of (**a**) sample A and (**b**) sample B at *V*_GS_ = −25 V and *V*_DS_ = 1100 V.

To further investigate the carrier behavior under high drain bias, two-dimensional off-state simulations were performed at *V*_GS_ = −25 V and *V*_DS_ = 1100 V. As shown in Figure 8, sample B exhibits a clear redistribution of the drain-side electrostatic potential compared with sample A. The change is mainly observed in the GaN channel and near the channel/buffer interface. This indicates that the step-graded AlGaN buffer modifies the electrostatic potential distribution beneath the channel under high drain bias. As shown in Figure 9, sample B also shows stronger electron depletion in the gate–drain region. The remaining electrons are more localized near the source-side channel. The electron population extending toward the underlying buffer is also reduced. These results indicate enhanced carrier confinement and reduced electron penetration under high drain bias. This behavior may contribute to the improved off-state blocking performance.

## 4. Conclusions

In summary, GaN HEMTs incorporating step-graded AlGaN buffer layers were demonstrated for enhanced off-state breakdown performance. The step-graded AlGaN buffers increased the 2DEG sheet density compared with the conventional GaN-buffer structure. Although the electron mobility decreased, the fabricated devices maintained comparable output currents. At approximately 1000 V, sample B exhibits an average leakage-current reduction of approximately 27.5% compared with sample A, while the average breakdown voltage increases from 1540 V to 1814 V, corresponding to an enhancement of approximately 17.8%. These results indicate improved high-voltage blocking capability in the step-graded AlGaN-buffer device. The step-graded AlGaN buffer introduces a higher electron-blocking barrier and a step-like band profile, while also enhancing vertical carrier confinement. Under high drain bias, the step-graded buffer also modifies the drain-side electrostatic-potential distribution and reduces electron penetration toward the underlying buffer. Dislocation-related states may also contribute to electron trapping or compensation under high-field conditions and thereby further suppress leakage transport.

## Figures and Tables

**Table 1:** Summary of Raman, HRXRD, and Hall measurement results for samples A–D.

Sample	Raman E_2_ (High) (cm^−1^)	XRD (002) (Arcsec)	XRD (102) (Arcsec)	*n*_s_ (cm^−2^)	*μ* (cm^2^/V·s)
A	572.4	106.7	416.8	1.09 × 10^13^	1817.3
B	573.1	170.9	1090.4	1.18 × 10^13^	1578.77
C	573.1	171.1	1045.0	1.19 × 10^13^	1555.17
D	573.1	168.9	1089.1	1.20 × 10^13^	1558.32

## Data Availability

The original contributions presented in the study are included in the article; further inquiries can be directed to the corresponding author.
